# Can natural variation in grain P concentrations be exploited in rice breeding to lower fertilizer requirements?

**DOI:** 10.1371/journal.pone.0179484

**Published:** 2017-06-26

**Authors:** Fanmiao Wang, James Douglas Morrison King, Terry Rose, Tobias Kretzschmar, Matthias Wissuwa

**Affiliations:** 1 Graduate School of Agricultural and Life Sciences, The University of Tokyo, 1-1-1, Yayoi, Bunkyo-ku, Tokyo, Japan; 2 Crop, Livestock and Environment Division, Japan International Research Center for Agricultural Sciences, 1–1 Ohwashi, Tsukuba, Ibaraki, Japan; 3 International Rice Research Institute (IRRI), Los Banos, Laguna, Philippines; 4 Southern Cross Plant Science, Southern Cross University, Lismore, NSW, Australia; Louisiana State University, UNITED STATES

## Abstract

Agricultural usage of phosphorus (P) is largely driven by the amount of P removed from fields in harvested plant matter as offtake needs to be balanced by P fertilizer application. Reducing P concentration in grains is a way to decrease P offtake and reduce P fertilizer requirements or soil P mining where insufficient P is applied. Our objective was to assesses the genotypic variation for grain P concentration present within the rice gene pool and resolve to what extent it is affected by environment (P supply) or associated with genetic factors. About 2-fold variation in grain P concentrations were detected in two rice diversity panels, however, environmental effects were stronger than genotype effects. Genome wide association studies identified several putative loci associated with grain P concentrations. In most cases this was caused by minor haplotype associations with high grain P concentrations while associations with reduced P concentrations were identified on chromosomes 1, 6, 8, 11 and 12. Only the latter type of locus is of interest in breeding for reduced P concentrations and the most promising locus was at 20.7 Mb on chromosome 8, where a rare haplotype that was absent from all modern varieties studied reduced grain P concentration by 9.3%. This and all other loci were not consistently detected across environments or association panels, confirming that genetic effects were small compared to effects of environment. We conclude that the genetic effects detected were not sufficiently large or consistent to be of utility in plant breeding. Instead breeding efforts may have to rely on small to medium effect mutants already identified and attempt to achieve a more pronounced reduction in grain P concentration through the introgression of these mutants into a single genetic background.

## Introduction

Agricultural productivity largely relies on continual replenishment of soil nutrients as nutrients are removed from the system in harvested produce. However, the amount of nutrients needed to be applied to fields to sustain high yielding crops can be reduced if the crop cultivars grown are efficient at using these nutrients to produce harvestable yield. This is particularly important for phosphorus (P) because the major source of P fertilizer, phosphate rock, is non-renewable and unevenly distributed worldwide [1, 2]. On the other hand, farmers in poor regions are often unable to apply adequate P fertilizer because of increasing prices and poor infrastructure, and thus crop yields in low-input systems are often limited by P availability. Breeding for P efficiency is therefore an important step towards developing sustainable cropping systems in both high yielding and low input scenarios [3].

Agricultural usage of P is to a large degree driven by the amount of P taken off fields in harvested plant matter. In cereal crops 60–85% of the aboveground P is typically found in the grains and is therefore removed at harvest [4, 5]. This leads to soil P mining and subsequently decreasing soil fertility in many poor regions where P offtake is not balanced by fertilizer addition [6, 7]. At the opposite end of the spectrum, excess of fertilizer application far beyond levels needed to replenished P offtake occurs in some regions [6], and erosion of such P-rich soil [8]. Furthermore, excessive P translocation to seeds may indirectly contribute to pollution because the major form of P stored in seeds, phytate, is very poorly absorbed by humans and other mono-gastric animals, thus increasing the P load in effluent and animal wastes [9].

Reducing grain P concentration via breeding has been proposed as a method of decreasing P offtake and subsequently reducing maintenance fertilizer requirements in high input systems or slowing the rate of soil P mining in low input systems [5, 9, 10]. In contrast to many earlier reports, recent studies have found that using seeds with low P concentrations (and content) may not have a negative impact on seedling vigor and grain yield [3, 11, 12], including rice seedlings derived from batches with P concentrations as low as 1 mg g^-1^ [11]. Typical P concentrations in seed batches obtained from farmers’ fields in South East Asia tend to be in the range of 2.0–2.5 mg g^-1^ [13], indicating that a large potential reduction in P offtake from field exists if a low-P seed trait could be developed through crop breeding [14]. However, our understanding of the regulation of P translocation and grain P loading during grain filling is limited. In a previous study on P transporter expression patterns during grain filling, there was no evidence that any particular P transporter was specifically responsible for grain P loading [15].

Attempts to alter grain P content though mutations have mostly focused on reducing phytate in the grain but in most cases low phytic acid (*lpa*) mutants simply balance reduced grain phytate with a proportional increase in inorganic P (Pi) [9]. In barley, the *Hvlpa1-1* mutant was an exception as the loss of sulfate transporter (*HvST*) activity lead not only to a reduction of phytate but also of total grain P concentrations and content of around 15% [16]. In rice, Zhao et al. (2016) [17] reported a loss of function in the putative sulfate transporter, *OsSULTR3;3*, altered grain metabolism and lead to a reduction of 22% in total grain P concentration across years. Additionally, an effect of 15–20% lowered grain P concentration was recently reported in studies with rice mutants of the SULTR-like Phosphorus Distribution Transporter (*SPDT*), which partly regulates the allocation of P between leaves and panicles [18].

While mutants may offer some scope to reduce grain P concentration, the extent to which reductions are possible seem limited to the around 20% with currently available mutants. To extend that range one may exploit natural genetic variation for grain P concentrations. It should be stressed that grain P concentration rather than content is of importance because the total P removed at harvest is the product of crop yield and grain P concentrations, whereas single grain content is subject to compensatory effects of other yield components (i.e. at equal grain yield larger seeds that may have higher P content would be balanced by reduced grain number). In studies that report grain P concentrations of different rice varieties over various sites and/or levels of P supply/availability, variation is typically in the range of 1–5 mg P g-1 [10, 19, 20]. A recent study examined to what extent this variation is due to genotype or environment effects and concluded that the environment is the dominant factor, with P concentrations between genotypes within sites ranging from 0.5–2.0 mg g^-1^ [21]. The degree to which genotypic variation in grain P concentrations is caused by differences in P remobilization to grains vs variation in new P uptake during the reproductive phase remains unknown. Previous research on determinant crops like wheat reported that the final grain P depends on re-translocation from vegetative organs [22]. However, using ^33^P isotope, Julia et al. (2016) [23] estimated that up to 70% of total grain P in the rice variety IR64 originated from P uptake post-flowering. While environment and G × E effects seem to be dominant factors, Vandamme et al. (2016c) [21] identified some rice genotypes including SANTHI SUFAID and DJ123 as having consistently below-average grain P concentrations across environments, which would make them potential donors for breeding programs. However, that study used a limited number of genotypes and to date it remains unknown to what extent genotypic variation for grain P concentration is present in the rice gene pool. Furthermore, quantitative trait loci (QTL) and linked markers that would allow efficient selection for a low grain P trait are currently unavailable, yet would be very desirable given the difficulty of classical breeding due to the strong effect of the environment on this trait and the fact that the trait cannot be assessed until post-harvest, slowing the breeding cycle.

In this study, our objective was to assess the natural variation for grain P concentrations present in the rice gene pool based on field experiment conducted at two levels of P supply. Our data from a previous study [24] indicated that the *japonica* subspecies of rice typically has higher grain P concentrations compared to *indica* rice (Table 1). Since our target was to identify possible donors and loci for reduced grain P concentrations, we focused on a diversity panel of 249 *indica* accessions. Based on the variation present within this panel our second objective was to identify putative loci contributing to grain P concentrations through a genome wide association study (GWAS).

**Table 1:** Phenotypic means, standard deviations and ranges of a broad rice association panel grown in at the JIRCAS subtropical research station in Ishigaki/Okinawa.

Sub population	Grain P concentration (mg g-1)		Grain yield (g plant-1)		Grain P content (mg)	
	Mean (STD)	Range	Mean (STD)	Range	Mean (STD)	Range
***Aus***	3.24 (0.30)	2.54–3.94	23.5 (3.64)	16.7–32.5	76.2 (14.4)	50.4–115
***Indica***	3.11 (0.36)	2.43–4.38	25.1 (3.32)	16.3–33.8	77.9 (14.3)	45.6–123
***TeJ***	3.39 (0.62)	2.65–4.72	25.5 (2.70)	21.5–31.6	87.2 (22.9)	66.6–149
***TrJ***	3.22 (0.40)	2.65–4.66	28.9 (5.06)	18.2–40.7	93.0 (20.0)	48.3–169
***All***	3.22 (0.38)	2.43–4.72	25.8 (4.49)	16.3–40.7	83.0 (18.0)	45.6–169

n = 54 for *aus*, 66 for *indica*, 16 for *temperate japonica* and 54 for *tropical japonica*, n = 219 for all.

## Materials and methods

### Field experiment

The main experiment was conducted with an *indica* rice diversity panel composed of 249 rice genotypes. The experimental site was a farmer’s field in the village of Pangil, the Philippines, about 50 km northeast of the International Rice Research Institute (IRRI). Plants were grown from January 2014 to May 2014 in either a -P plot to which no P fertilizer had been applied for more than 10 years (available P: 3.0 mg kg^-1^ soil (Bray)) or a +P plot receiving 50 kg of P_2_O_4_ ha^-1^ annually (available P: 14.0 mg kg^-1^ soil (Bray)). Nitrogen (N) and potassium (K) fertilizer was applied as recommended: 50 kg N ha^-1^ and 50 kg K ha^-1^ basally before final land preparation, plus an additional dose of 50 kg N ha^-1^ as urea at late tillering. Within P treatment plots, two replicates for each genotype were arranged in a random complete block design. Each plot was 3 m long with two rows of 16 plants at 20 cm intervals per row. Biomass and grain yield were measured at maturity and used to calculate harvest index (HI = grain yield/total aboveground biomass). Straw and grain from five plants per plot were pooled and ground to powder for determination of tissue P concentrations. The P harvest index (PHI = grain P content/total aboveground biomass P content) was then calculated.

Additionally, we used data on P concentrations in seeds utilized for an experiment described in detail in [24]. A total of 219 accessions of the broad rice association panel described in detail by [25] had been grown in 2m rows at the Japan international Research Center for Agricultural Sciences (JIRCAS) subtropical research station in Ishigaki/Okinawa between February and June 2011. The field had been fertilized with 50 kg ha^-1^ NPK basally before final land preparation, plus an additional dose of 50 kg N ha^-1^ as urea at late tillering. After harvest of straw and grains, respective yields were calculated and grain ground for further processing as above.

### Tissue phosphorus analysis

Sample digestion and P measurements were done at JIRCAS, Tsukuba, Japan. Ground grain and straw subsamples (about 100 mg grain and 250 mg straw) were pre-digested in 8 ml of nitric acid in 50 ml Digitubes (Acid digestion tubes, GL Sciences) overnight, followed by digestion at 105^°^C for 2 h in a block digester. To fully oxidize samples, 2 ml of hydrogen peroxide was added to the solution and heated at 105^°^C for a further 2 h. After cooling, the digested solution was diluted to 50 ml with distilled water and filtered through ADVANTEC No. 6 filter paper. Phosphorus concentrations in straw were measured by the molybdenum blue method [26]. Absorptions were determined using a microplate reader with the following reaction mix in each well: 25 μl of Molybdenum reagent, 10 μl of digested sample solution and 190 μl of distilled water. Absorption at 840nm wave length was read after 1 h incubation at 37^°^C to ensure complete reaction. Phosphorus concentrations in grain samples were quantified by ICP-AES (ICPE-9000, Shimadzu) because the colorimetric method underestimated P concentrations in a reference grain sample of known P concentration.

### GWAS and statistical analysis

The 700K SNP genotyping data [27] and the software Tassel 5 were used for the association study. The SNP data were modified as follows: heterozygous SNPs were first set to missing values. They were further filtered using 2% as the minimum allele frequency and 85% as the minimum count. In the end, 261K SNPs remained for the analysis. Principal component (PC) analysis was performed using an R package (R version 3.1.1) called Genome Association and Prediction Integrated Tool (GAPIT) to control for population structure and the obtained PC data was imported to Tassel for GWAS analysis. As the emphasis of the experiment shifted (see discussion) we attempted to minimize the type-II error (false-negatives) and selected peaks with thresholds of 1.0E-04 (for mixed linear model, MLM) or 1.0E-05 (for general linear model, GLM) for further detailed investigation. Using linkage disequilibrium (LD) analysis in HaploView 4.2, QTL regions were defined by markers being linked to the peak marker with an R^2^ ≥ 0.65 (within 80–360 Kb region around the peaks). For the haplotype analysis markers in that LD block with low P value in GWAS were included. Accessions with missing yield data or a HI below 0.30 in both replicates were excluded in the genome wide association analysis to avoid potentially confounding effects (insufficient dilution with low grain yields). In total 150 accessions were included in the GWAS using the *indica* diversity panel. In the case of the broad association panel the 44K SNP dataset [25] was used and associations detected using Tassel 5 with 219 accessions of the *indica*, *aus* and *tropical japonica* subpopulations.

## Results

### Phenotypic traits and correlations between traits

Treatment mean values for the *indica* rice diversity panel are shown in Table 2. The average total aboveground biomass in low P soil was 31.8 g plant^-1^, around 75% of that in high P treatment (41.4 g plant^-1^). Two thirds of the decrease in biomass was due to reduced straw weight while grain yield was only reduced by 16%. This led to an increase in HI from 47.9 at high P to 52.7 at low P. The average straw P concentration was 0.51 mg g^-1^ in low P soil and this doubled in the high P treatment. Grain P concentration also increased, but by less than 50% from 2.5 mg g^-1^ in low P to 3.6 mg g^-1^ in high P. The combination of higher biomass and P concentrations resulted in a total plant P content almost twice as high in the high P treatment. On the other hand, the strong decrease in shoot P concentrations in the low P treatment led to a significantly higher PHI of 84.8 compared to 76.5 in high P, indicating proportionally more P was partitioned to seeds under limiting compared to sufficient P supply. For the *indica* diversity panel, the PHI varied from 57–90% under high P conditions, while under P deficiency it varied from 71–94%.

**Table 2:** Phenotypic means and the 95% confidence interval of a rice *indica* diversity panel grown in the fields with +P and–P treatments.

* *	-P	95% C.I.	+P	95% C.I.
**Straw weight (g plant-1)**	15.1	14.4–15.8	21.5	20.6–22.4
**Grain yield (g plant-1)**	16.7	16.0–17.4	19.8	19.1–20.6
**Biomass (g plant-1)**	31.8	30.5–33.1	41.4	39.9–42.8
**Harvest Index**	52.7	52.0–53.4	47.9	47.0–48.8
**Straw P concentration (mg g-1)**	0.51	0.48–0.54	1.01	0.97–1.05
**Straw P content (mg)**	7.26	6.91–7.62	21.5	20.3–22.7
**Grain P concentration (mg g-1)**	2.5	2.45–2.55	3.64	3.58–3.70
**Grain P content (mg)**	41.6	39.8–43.4	70.1	67.0–73.2
**Total P content (mg)**	48.8	46.9–50.7	91.6	87.9–95.3
**P Harvest Index**	84.8	84.1–85.5	76.5	75.5–77.5

n = 193 for straw weight, grain yield, biomass, harvest index, straw P concentration and straw P content; n = 179 (-P) and n = 180 (+P) for grain P concentration, grain P content, total P content and P harvest index.

**Fig 1:**
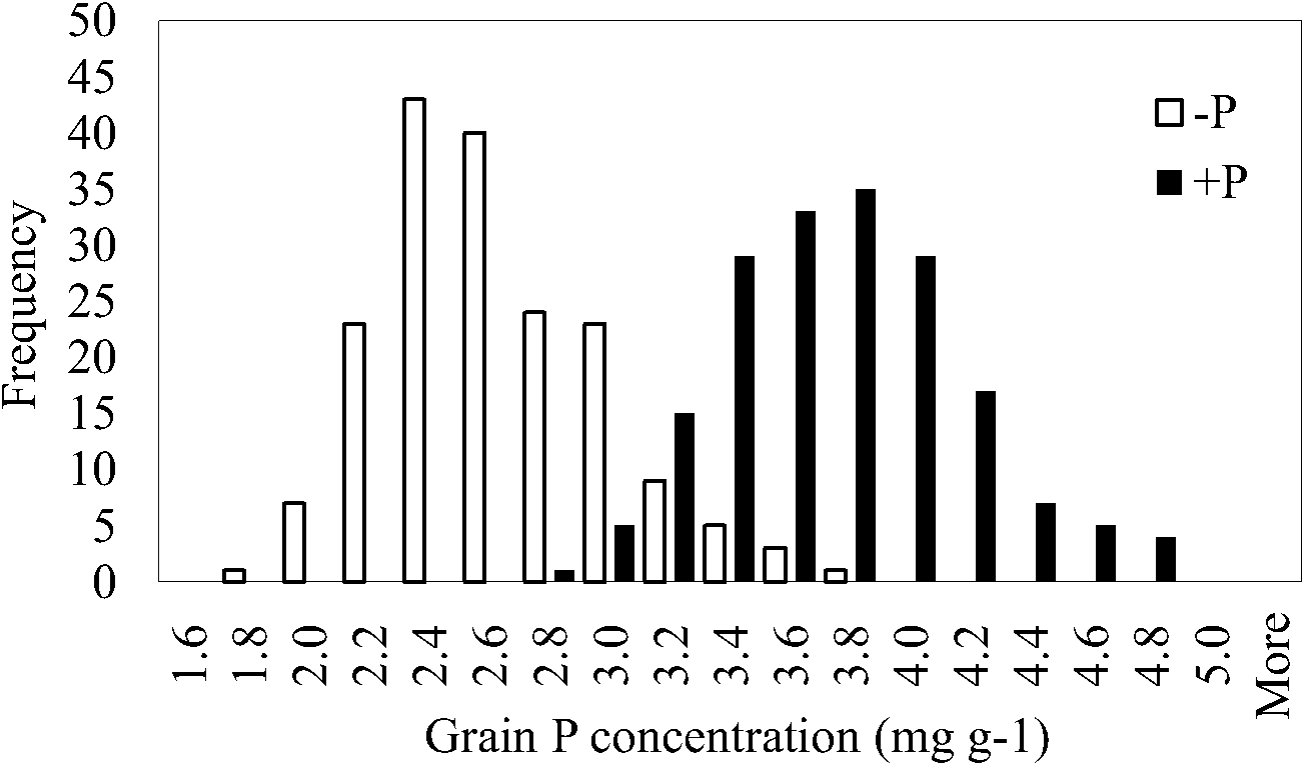
Frequency distribution for grain P concentrations of a rice *indica* panel grown in the unfertilized (-P, n = 179) and P fertilized fields (+P, n = 180).

Grain P concentrations showed considerable variation in both treatments, ranging from 1.81–3.75 mg g^-1^ in low P and 2.77–4.78 mg g^-1^ in high P (Fig 1). In the low P treatment, grain P concentration of genotypes ranged from 28% below the mean to 50% above the mean. This compares to a range between 24% below the mean to 31% above the mean in the high P treatment. The variation in the high P treatment was very similar to the results for the broad rice association panel (Table 1) where grain P concentrations varied from 2.43–4.38 mg g^-1^ in the *indica* subpopulation compared to 2.43–4.72 mg g^-1^ across all subpopulations.

In the high P treatment at Ishigaki/Okinawa, the lowest grain P concentration (2.77 mg g^-1^) was found in accession SUDUWEE while VANDANA had the highest (4.78 mg g^-1^). WAS 203 and JINLING had the lowest (1.81 mg g^-1^) and highest (3.75 mg g^-1^) grain P concentrations in the low P treatment. Despite the lack of correlation between grain P concentration across P treatments (r = 0.07), some consistency was observed for several genotypes. Eight genotypes had grain P concentrations one standard deviation below the treatment mean in both treatments: WAS 203, NIONOKA, THAPACHINIYA, ANGIFOTSY 685, PEH-KUH-TSAO-TU, HONG MI DONG MAO ZHAN, PSBRC 18, CICA 8. Consistently high (at least one standard deviation above the mean) grain P concentrations were detected in six genotypes: TOKAMBANY, KHAO DAW TAI, NCS 130, JINLING 78, SOM CAU 70 A and KUMBI. For genotypes with low grain P concentrations in the low P treatment (29 genotypes in total), eight had above average grain P concentrations in high P conditions, indicating P supply had a considerable effect on determining the grain P concentration. Of the 30 accessions that had the lowest grain P in the high P treatment, nine had above average grain P concentration in low P conditions.

To investigate relationships between all studied traits, a correlation analysis was conducted within each P treatment. Generally, very similar trends were observed in both P treatments. Grain and straw biomass showed equally strong correlations to total biomass but only weak correlations with HI (Table 3). The correlations between straw P concentration and biomass were negative but weak in both treatments (-0.45 in low P and -0.23 in high P). The strongest correlations of straw P concentrations were with PHI (-0.80 under low P and -0.69 with high P). Grain P concentrations on the other hand, were not correlated with PHI as one may have expected, and only marginally correlated with grain yield (r = -0.18 or -0.10 in low P and high P, respectively), indicating that higher grain yield did not automatically lead to a dilution of grain P concentrations. Grain P content was tightly correlated with grain yield in both P treatments whereas the correlation between straw biomass and straw P content was weak (0.47) in the low P treatment and only moderate (0.72) in the high P treatment.

**Table 3:** Correlation coefficients between traits measured in a rice *indica* diversity panel grown either in a +P (above diagonal) or–P field (below diagonal).

	StW	GY	BM	HI	StPconc	GPconc	StP	GP	TP	PHI
**StW**	***0*.*47***	0.57	0.90	-0.44	-0.14	-0.03	0.72	0.56	0.71	-0.22
**GY**	0.76	***0*.*11***	0.87	0.44	-0.28	-0.10	0.27	0.92	0.87	0.45
**BM**	0.94	0.94	***0*.*31***	-0.03	-0.23	-0.07	0.57	0.82	0.89	0.10
**HI**	-0.40	0.27	-0.07	***0*.*17***	-0.13	-0.04	-0.44	0.39	0.18	0.75
**StPconc**	-0.35	-0.48	-0.45	-0.15	***0*.*17***	0.19	0.56	-0.21	0.00	-0.69
**GPconc**	-0.04	-0.18	-0.12	-0.17	0.40	***0*.*07***	0.10	0.27	0.26	0.10
**StP**	0.47	0.18	0.35	-0.44	0.59	0.28	***0*.*11***	0.31	0.59	-0.65
**GP**	0.74	0.89	0.87	0.16	-0.30	0.27	0.33	***0*.*11***	0.95	0.47
**TP**	0.77	0.85	0.86	0.05	-0.15	0.31	0.51	0.98	***0*.*17***	0.18
**PHI**	0.11	0.51	0.33	0.59	-0.80	-0.08	-0.65	0.45	0.27	***-0*.*01***

Values in the diagonal are correlations within traits across P treatments. StW, GY, BM, HI, StPconc, GPconc, StP, GP, TP and PHI stand for straw weight, grain yield, biomass, harvest index, straw P concentration, grain P concentration, straw P content, grain P content and total P content, respectively. P<0.05 at r = 0.13 (+P), P<0.05 at r = 0.12 (-P), P<0.05 at r = 0.17 (for across-P level correlations); n = 193 for StW, GY, BM, HI, StPconc and StP; n = 179 (-P) and n = 180 (+P) for GPconc, GP, TP and PHI.

### GWAS for grain P concentration and related traits

**Fig 2:**
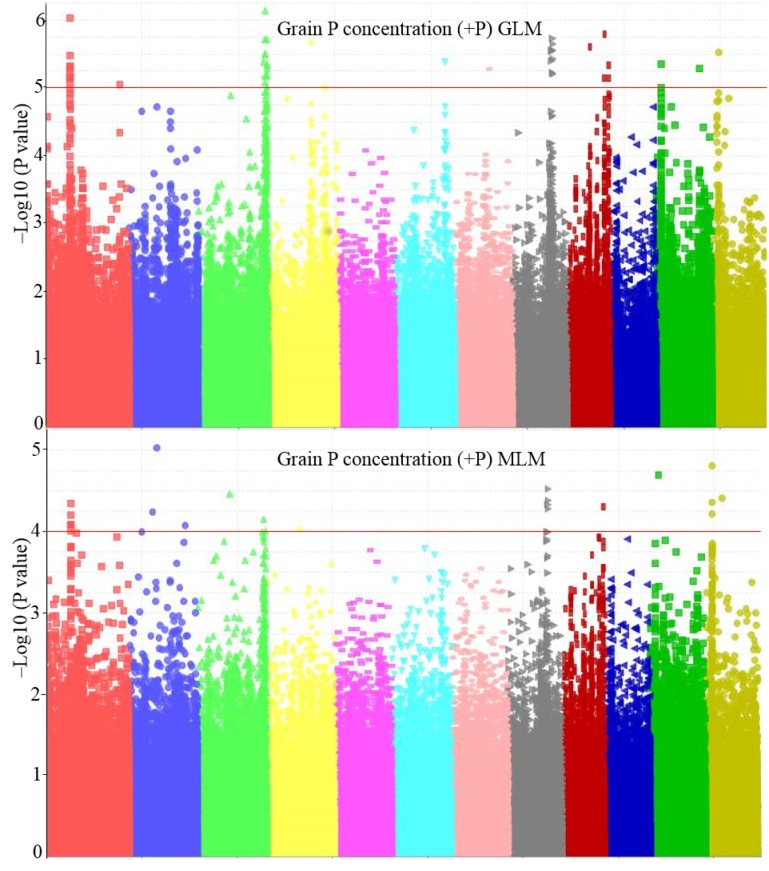
Manhattan plot of grain P concentration (+P) using general linear model (GLM) and mixed linear model (MLM).

We employed two statistical models in our GWAS analysis, GLM and MLM. Both analysis included three principal components describing population structure whereas MLM additionally included a kinship coefficient matrix to account for the genetic resemblance between individuals. The population structure within the *indica* panel was not very pronounced as evident from principal components explaining only a small portion of the variance present (7%, 6% and 4% for PC 1–3, respectively, S1 Fig). GLM overestimated the significance of effects as can be seen in the QQ plots whereas effects were underestimated in MLM (S2 Fig). Results of both analyses are shown as Manhattan plots in Fig 2 and peaks were considered for further in-depth analysis if probabilities exceeded thresholds of 1.0E-04 (MLM) or 1.0E-05 (GLM) (Table 4).

**Table 4:** Loci detected as being associated with grain P concentration in the +P and–P treatments as listed for peak tip markers.

	QTL	SNP ID	Chromo some	Position (Mb)	MLM	GLM	MAF	Minor allele effect (%)		IR8	IR36	IR64	IR72	PSBRC18
								GP conc.	TP					
**+P**	1	SNP-1.12656929.	1	12.6	4.5E-05	9.3E-07	0.38	**-7.9**	-8.3	↓	↓	M	↓	↓
	2	SNP-1.38440214.	1	38.4	7.7E-04	8.8E-06	0.17	12.3	4.6	—————M—————				
	3	SNP-2.12256792.	2	12.2	5.8E-05	4.1E-04	0.03	18.9	-14.6	—————M—————				
	4	SNP-2.14422340.	2	14.4	9.4E-06	1.9E-05	0.03	23.1	42.1	—————M—————				
	5	SNP-2.29388834.	2	29.3	8.4E-05	1.1E-04	0.10	13.5	11.9	—————M—————				
	6	SNP-3.16821855.	3	16.8	3.4E-05	1.3E-05	0.03	23.5	18.7	—————M—————				
	7	SNP-3.34589288.	3	34.5	7.0E-05	7.3E-07	0.12	11.5	-3.6	—————M—————				
	8	SNP-3.35607907.	3	35.6	9.7E-05	4.5E-06	0.08	13.7	5.7	—————M—————				
	9	SNP-4.17094445.	4	17.2	9.5E-05	1.2E-03	0.04	16.1	16.1	—————M—————				
	10	SNP-4.21777790.	4	21.9	4.9E-04	2.2E-06	0.32	10.9	0.6	—————M—————				
	11	SNP-4.29018566.	4	29.2	4.6E-03	1.0E-05	0.07	5.1	16.0	—————M—————				
	12	SNP-6.26075242.	6	26.0	3.2E-04	4.2E-06	0.48	**-8.9**	-8.9	↓	↓	↓	↓	↓
	13	SNP-7.17796564.	7	17.7	7.7E-04	5.2E-06	0.31	9.3	4.6	—————M—————				
	14	SNP-8.20742013.	8	20.7	4.6E-05	1.9E-06	0.06	**-9.6**	7.7	—————M—————				
	15	SNP-8.21117439.	8	21.1	3.0E-05	2.3E-06	0.06	**-6.3**	13.2	—————M—————				
	16	SNP-9.11782248.	9	11.7	3.5E-04	2.5E-06	0.47	10.2	3.6	—————M—————				
	17	SNP-9.19360989.	9	19.3	1.2E-04	1.6E-06	0.23	4.4	11.3	—————M—————				
	18	SNP-9.21513573.	9	21.5	5.0E-05	3.3E-06	0.23	10.3	-3.2	—————M—————				
		SNP-9.21541974.	9	21.5	1.3E-04	4.6E-06	0.27	10.0	-0.6	—————M—————				
	19	SNP-11.2640192.	11	2.6	2.5E-04	4.4E-06	0.39	**-6.5**	3.5	—————M—————				
	20	SNP-11.4316418.	11	4.3	2.0E-05	3.9E-05	0.06	17.0	25.9	—————M—————				
	21	SNP-11.21996145.	11	22.4	4.1E-04	5.2E-06	0.41	5.2	7.6	M	↓	M	M	M
	22	SNP-12.3476040.	12	3.4	1.6E-05	3.0E-06	0.28	**-4.6**	-5.1	—————M—————				
	23	SNP-12.8667207.	12	8.6	3.9E-05	1.4E-05	0.33	**-6.1**	-8.8	—————M—————				
**-P**	1	SNP-2.11382641.	2	11.3	9.7E-05	5.3E-05	0.03	29.3	-15.7	—————M—————				
	2	SNP-2.24241551.	2	24.2	3.5E-06	2.0E-07	0.02	38.5	0.8	—————M—————				
	3	SNP-4.30293581.	4	30.5	1.4E-04	3.0E-07	0.07	25.8	22.3	—————M—————				
	4	SNP-4.34334276.	4	34.5	8.8E-05	1.0E-04	0.03	27.9	-0.1	—————M—————				
	5	SNP-9.20149491.	9	20.1	1.6E-04	2.8E-07	0.14	19.5	27.3	—————M—————				

MLM: mixed linear model; GLM: general linear model; MAF: minor allele frequency; GPconc: grain P concentration; TP: Total P; Minor allele effect was calculated as: (phenotypic mean of minor allele–phenotypic mean of major allele) / phenotypic mean of major allele × 100%; “—M—” denotes accessions have major allele; “↓”denotes accessions have minor allele.

The weak correlation between grain P concentration of both P treatments (r = 0.07 –see above) was further confirmed by the GWAS, since none of the peaks identified were common to both treatments (Table 4). Combining the MLM and GLM data, we identified 23 QTLs (24 peaks) located on all chromosomes except for chromosomes 5 and 10 in the high P treatment and 5 peaks in the low P treatment. Minor allele frequencies and minor allele phenotype effects on grain P concentration and total P content relative to the phenotypic mean of the major allele are shown in Table 4. We also listed the alleles for the five most popular IRRI varieties (IR8, IR36, IR64, IR72, PSBRC18).

Most (22 out of 29) of the minor alleles had effects of increasing grain P concentration (Table 4). Thus, genotypes with the more common allele had average or below average grain P concentrations and scope to reduce grain P concentrations using these peaks does not exist. Some of the hallmark rice varieties developed at IRRI had mostly major alleles and below average grain P concentrations (3.31, 3.41, 3.46, 3.17 and 3.19 mg g^-1^ for IR8, IR36, IR64, IR72 and PSBRC18, respectively). Peaks of more interest were those with minor alleles contributing to a reduction of grain P concentrations below the treatment average, which was observed only in the high P treatment for peaks on chromosomes 1, 6, 8, 11 and 12. The peak at 20.7 Mb on chromosome 8 had a very low minor allele frequency of 0.06 and showed the biggest reduction in grain P concentrations of nearly 10% for the minor allele. Other reductions for minor alleles were in the range of 9% (26.0 Mb on chromosome 6) to as low as 4.6% on chromosome 12. For the peaks reducing grain P concentration we indicated whether the major IRRI varieties belonged to the haplotype reducing or increasing grain P concentrations. For peaks on chromosome 8, 11 and 12 they belonged to the major haplotype and may thus benefit from the introduction of alleles reducing grain P concentrations. Minor alleles reducing grain P concentrations on chromosomes 1 (12.6 Mb) and chromosome 6 (26.0 Mb) were very common (MAF 0.38–0.48) and already present in IRRI varieties.

We further tested whether peaks identified for grain P concentrations co-localized with peaks for other traits of importance like grain yield, total P and PHI. We only analyzed the high P treatment data because in the low P treatment, minor alleles contributed to high grain P concentration and were therefore of no practical relevance. Generally, the GWAS study for the three traits did not share peaks with grain P concentration (S3 Fig) as expected given the weak correlations between grain P concentration and the traits analyzed (r = 0.10 for grain yield and PHI; r = 0.26 for total P). For 17 out of the 22 peaks where the minor allele led to increased grain P concentration, the minor allele also had higher total P. This was highlighted by the peak at chromosome 2 (14.4Mb): a 23% increased grain P concentration was accompanied with a 42% increase in total P, indicating that grain P concentrations increase as more P is taken up. The final grain P concentration also depends on the proportion of P translocated to grain at harvest (PHI), however, no common significant peaks were detected.

For the broad association panel, several associations were detected for grain P concentrations using MLM with a threshold of 1.0E-4 and in all cases these were subpopulation-specific. Four peaks on chromosomes 2, 4, 5 and 6 were *indica*-specific, while one peak on chromosome 11 was *japonica*-specific (Table 5). The minor alleles for all loci had effects of increasing grain P concentrations by 23–34% for *indica* and 14–20% for *tropical japonica*. The *indica* peaks had very low MAFs and effects were either due to only two or four accessions having the rare SNP alleles leading to increased grain P concentrations.

**Table 5:** Loci detected as being associated with grain P concentration in subpopulations.

QTL	Chr	Interval (Mb)	MLM	MAF	Minor allele effect on GPconc (%)	sub population
1	2	30.2–31.7	2.66E-06	0.03	32.0	*indica*
	4	10.6–12.6	2.66E-06	0.03	32.0	*indica*
2	5	2.89–2.94	1.35E-05	0.06	22.9	*indica*
3	6	10.7–10.8	2.30E-07	0.03	33.8	*indica*
4	11	6.61	3.63E-06	0.20	19.9	*TrJ*

MLM: mixed linear model; MAF: minor allele frequency; GPconc: grain P concentration; Minor allele effect was calculated as: (mean of minor allele–mean of major allele) / mean of major allele × 100%.

### Haplotype analysis

For practical breeding purposes, positive alleles that are not already present in most rice accessions, or at least not in very commonly grown rice varieties, are of particular interest, so particular attention was paid to minor alleles. Since all the minor alleles in the analysis for the broad association panel had the effects of increasing grain P concentration, we did not include them in the haplotype analysis.

**Fig 3:**
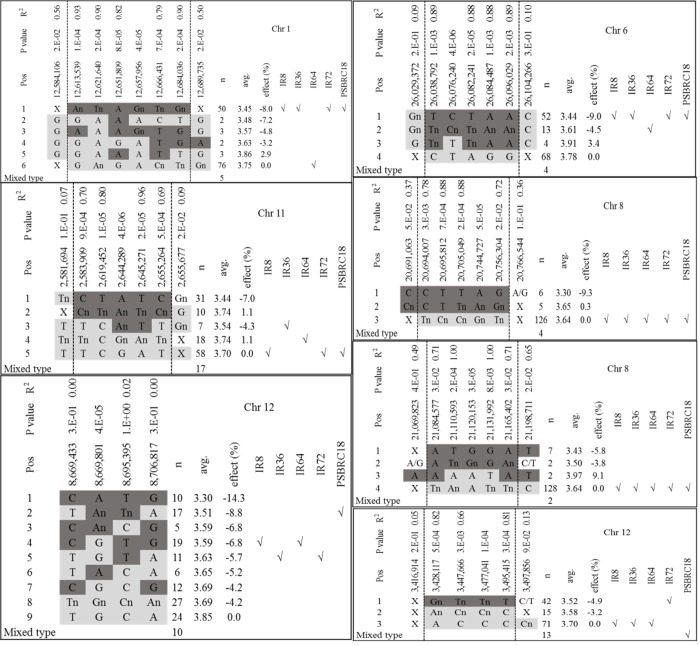
Haplotypes analysis for loci associated with grain P concentration on chromosome 1, 6, 8, 11and 12. Linkage disequilibrium (LD) blocks are within the two dashed lines based on the R^2^ values from the LD analysis, with flanking SNPs on both sides. SNP alleles reducing grain P concentrations are highlighted in dark grey while ‘negative’ alleles increasing grain P concentrations are highlighted in light grey, “X” denotes mix of major, minor and missing alleles, “√” indicates which haplotype the accessions belong to. See S4–S10 Figs for more information of LD analysis.

In the *indica* diversity panel minor alleles reduced grain P concentrations at peaks on chromosomes 1, 6, 8, 11 and 12 (Table 4) and haplotype patterns surrounding these peaks were investigated for markers in the LD blocks (S4–S10 Figs). On chromosome 1 several rare intermediate haplotypes existed in addition to the major and the rather common minor haplotype, which reduced grain P concentrations by 8% relative to the major haplotype (Fig 3). Most IRRI varieties with the exception of IR64 belonged to the minor haplotype, which was also the case for chromosome 6. Two rare haplotypes (MAF = 4.3% and 5.0%) on chromosome 8 (20.7 Mb and 21.1 Mb) reduced grain P concentration by 9.3% and 5.8% respectively compared with the phenotypic mean of the major haplotypes that included the IRRI varieties. And a common minor haplotype on chromosome 11 had a 7% lower grain P concentration than the major haplotype. An even bigger phenotypic effect was detected for the minor haplotype at 8.6 Mb on chromosome 12 (-14.3%); however, a larger number of intermediate haplotypes was detected and IRRI varieties belonged to these and would thus not benefit by a full 14.3% reduction in grain P concentration from introgression of this locus (Fig 3). In addition, the LD analysis (S10 Fig) indicated the peak SNP at 8.6 Mb on chromosome 12 was not linked to any other SNPs. The smallest haplotype effect was detected on chromosome 12 (3.4 Mb) where a rather common minor haplotype (frequency of 30%) reduced grain P concentrations by only 4.9%.

Haplotype effects on reduction of grain P concentration were often closed to single minor SNP effects (Table 4 and Fig 3). The biggest difference (14.3 vs 6.1%) was observed for the locus on chromosome 12 (8.6 Mb). This discrepancy was due to the presence of intermediate haplotypes containing minor alleles at some but not all SNP positions within the haplotype block.

## Discussion

Lowering grain P concentration will reduce P removal from fields at harvest, which in turn will reduce the maintenance P fertilizer application needed to balance offtake. This can be illustrated with an example as follows: Assuming that the yield achieved in a farmer’s field is 5000 kg and the grain P concentration is 3 mg g^-1^, this will lead to 15 kg of P being removed from the field that must be balanced by adding at least the same amount of P fertilizer to the field. If the grain P concentration can be reduced to 2 mg g^-1^, only 10 kg of P will be removed and 1/3 less P fertilizer would be needed to balance the offtake. Thus theoretical savings in terms of fertilizers are proportional to the reduction in grain P concentrations that can be achieved if the P in crop residue is as agronomical effective as P added in synthetic fertilizer or manure.

Here we evaluated whether genotypic variation for this trait is present within the rice gene pool and to what extent it can be associated with genotypic variation, by investigating P concentrations in seeds obtained from two rice diversity panels grown in the field either in the Philippines or Japan. Roughly two-fold variation existed among genotypes at each location and P treatment but no major QTL consistently explaining a large portion of the variation across environments and P treatments was detected in our GWAS analysis. Where allelic effects were large in single environments (more than 20%) they were due to the presence of very rare alleles that increased rather than decreased grain P concentrations. Such alleles are obviously of no utility in attempts to breed varieties with reduced grain P concentrations. It is not unlikely that such rare alleles may represent false positives, however, this was not further investigated since they are of no practical relevance. To further probe for loci reducing grain P concentrations and to reduce type-II error (false negative), we have employed less stringent detection thresholds and this allowed us to detect loci for which minor alleles reduced grain P concentrations, albeit with reductions only in the range of 5–10%. The most promising locus is at 20.7 Mb on chromosome 8 and a rare haplotype that is absent from all modern varieties studied reduced grain P concentration by 9.3%. Six genotypes in the *indica* panel have this minor allele, five of which origin from Madagascar (LOHAMBITRO 224, SOMIZY, TOKAMBANY663, TSIPALA FOTSY 1883, MAMORIAKA 114) and one from China (CHANG CH’SANG HSU TA). On average, these six genotypes have P uptake of 103 mg g^-1^, 13.8% higher than the average for the common haplotype, indicating their low grain P concentration is not simply a reflection of poor P uptake.

### Factors influencing grain P concentrations

The field experiment in the Philippines included two levels of P supply and the P supply effect was far more pronounced than the genotype effect (62% of the total variation in that experiment was due to the factor P, data not shown). Similarly, strong effects of the environment on rice grain P concentrations were reported in a G × E study across 41 environments in Africa and Asia [21]. Within a given environment or field, grain P concentrations will be a function of P taken up (TP, excluding P in roots), the proportion of P translocated to grain (PHI), and grain yield (GY). This can be confirmed based on experimental mean values in Table 2: 48.8 mg P × 0.848 × 1/16.7g = 2.48 mg P g^-1^. Thus, one would expect grain P concentrations to increase with increasing aboveground P content and PHI, and to decrease in higher yielding varieties. Interestingly, the correlation analysis shown in Table 3 does not confirm strong factor effects (r = 0.08 to 0.31) within P treatments. One possible conclusion is that grain P concentration is an independent trait, which is of interest from a breeding perspective as this would mean it should be possible to alter (reduce) it without affecting other plants traits such as grain yield or harvest index. This would be supported by findings of Stangoulis et al. (2007) [28] that grain P is under independent genetic control to a degree, and of Rose et al. (2010) [10] reporting an equally low correlation between GY and grain P concentration.

Alternatively, one may hypothesize that the low single-factor correlations can be the result of opposing effects negating each other. We therefore employed a multivariate approach to shed further light on this issue. The ‘best subset regression’ confirmed that individual factors had very low correlations with grain P concentrations (Table 6), however, a model using grain yield and total P together explained 54% (79% in -P) of the variation of grain P concentrations in the +P treatment and this increased to 90% (same in -P) with the addition of stem P content. Partial correlations with grain P concentrations, when controlled for variation in grain yield, confirmed a strong positive effect of P content on grain P concentrations (r = 0.73 and 0.88 in +P and -P respectively) (S1 Table). Similarly, when controlled for P content, partial correlations with grain yield increased to r = -0.71 (+P) and r = -0.87 (-P). In both cases PHI did not affect grain P concentrations (S1 Table) and this may have been a consequence of low variation for this trait (CV of 5.9 and 8.9 for +P and -P, respectively). The influence of both grain yields and total aboveground P accumulation on grain P concentrations suggests care needs to be taken when assessing the value of any QTL mapped for grain P concentration. For example, Blair et al. (2009) [29] mapped a QTL for grain P concentration in common bean to the same position as a QTL previously identified for adventitious rooting. Given that adventitious rooting in common bean is linked to higher P uptake from soil, it is highly likely that the grain P concentrations were directly linked to P uptake in this instance. Thus, while a number of QTLs for grain P concentration have been mapped in rice [28], in the absence of yield and P uptake data in that study it is difficult to ascertain the merit of any of the QTLs mapped.

**Table 6:** Best subset regression models for grain P concentrations and variables total P content (TP), grain yield (GY), straw P content (StP), and P harvest index (PHI) or straw P concentrations (StPconc).

**TP**	**GY**	**StP**	**PHI**	**R**^**2**^
+P treatment				
✓				0.07
	✓			0.03
		✓		0.01
			✓	0.01
✓	✓			0.54
✓	✓	✓		0.90
✓	✓	✓	✓	0.91
**TP**	**GY**	**StP**	**StPconc**	**R**^**2**^
-P treatment				
✓				0.10
	✓			0.03
		✓		0.09
			✓	0.17
✓	✓			0.79
✓	✓	✓		0.90
✓	✓	✓	✓	0.91

Model R^2^ are shown for individual variable regressions with grain P concentrations and best fit models that include two to four variables simultaneously (n = 293 for +P and 301 for -P treatments.

One additional reason why the proportion of P partitioned to grains (PHI) was not as influential as originally hypothesized could be related to the recent finding that rice variety IR64 took up as much as 70% of its total aboveground P post-anthesis [23] and that up to 80% of total grain P originated from post-anthesis uptake. These findings together with the poor association of PHI with grain P concentrations imply that breeding strategies aimed at reducing grain P concentrations will have to address the high rate of late P uptake possibly common in rice [10, 30], rather than focusing chiefly on re-translocation [31]. If targeting a reduction in late P uptake is not an acceptable strategy, one may have to find ways to deviate the late influx of P away from the panicle. The recently identified nodal rice transporter (*SPDT*) that determines what proportion P is allocated to the panicle [18] provides evidence that deviation of P fluxes away from the panicle is indeed a valid strategy to reduce grain P as knockout mutants of the *SPDT* transporter showed 20% reduced grain P concentrations.

While P uptake and translocation from vegetative tissues are key determinants of grain P concentrations, the process of loading P into grains from vascular tissue is equally critical, and unfortunately very little is understood about this process at the molecular level. An earlier study was unable to identify any P transporter with expression profiles consistent with a specific role in grain P loading [15], whereas a rice sulfate transporter (*OsSULTR3;3*) appears to be involved in this step and disruption of this gene leads to reduced concentrations of total grain P (19–28%) and of grain phytate (35–45%) [17]. Barley mutants with disruption to the homologue of *OsSULTR3;3* also have lower phytate and grain P concentrations [16]. Greater resolution of the key physiological and molecular processes involved in loading of P into grains may provide additional options to further reduce grain P concentrations through breeding.

### A breeding strategy for reduced grain P concentrations

The absence of clear associations with strong contributions to the desired low P phenotype in presence of large and normally distributed phenotypic variation suggests that grain P concentration is a rather complex quantitative trait, where many loci make small contributions. Pyramiding of several small-effect QTL is one option to make progress in breeding for such traits, however, it may be worthwhile considering whether a genomic prediction approach might yield faster and more robust results in a breeding program than a QTL introgression approach. For complex traits that show strong G × E such as yield genomic selection has made recent advances in rice breeding [32]. Since yield and grain P are apparently independent traits, the same training–prediction pipelines and models that are currently being established to drive yields up, could be employed to drive grain P concentrations down simultaneously. Such a strategy, however, would depend on a simple, high throughput phenotyping protocol for a reliable assessment of grain P concentrations.

While a genomic prediction approach seems feasible once high-throughput grain P analysis has been established, the most promising short-term option appears the utilization of already identified mutants in rice breeding. With effects of individual mutants in the range of 10–20% it remains to be seen to what extent grain P concentrations can be reduced by pyramiding two or three mutants in a single recipient cultivar.

## Supporting information

S1 Fig Variances explained by ten principal components using R package GAPIT. (TIF)

S2 Fig QQ plot of grain P concentration (+P) using general linear model (GLM) and mixed linear model (MLM). (TIF)

S3 Fig Manhattan plot of grain yield, total P and PHI in +P treatment using mixed linear model. (TIF)

S4 Fig Linkage disequilibrium (LD) analysis for loci associated with grain P concentration at 12.51–12.87 Mb on chromosome 1. Black arrows indicate peak tip SNPs and dashed arrows indicate SNPs included in haplotype analysis (listed in Fig 3). Numbers in boxes indicate R^2^ values between each two markers. Dark black boxes without numbers have R^2^ = 1.
(TIF)

S5 Fig Linkage disequilibrium (LD) analysis for loci associated with grain P concentration at 25.87–26.13 Mb on chromosome 6. Black arrows indicate peak tip SNPs and dashed arrows indicate SNPs included in haplotype analysis (listed in Fig 3). Numbers in boxes indicate R^2^ values between each two markers. Dark black boxes without numbers have R^2^ = 1.
(TIF)

S6 Fig Linkage disequilibrium (LD) analysis for loci associated with grain P concentration at 20.67–20.75 Mb on chromosome 8. Black arrows indicate peak tip SNPs and dashed arrows indicate SNPs included in haplotype analysis (listed in Fig 3). Numbers in boxes indicate R^2^ values between each two markers. Dark black boxes without numbers have R^2^ = 1.
(TIF)

S7 Fig Linkage disequilibrium (LD) analysis for loci associated with grain P concentration at 21.06–21.17 Mb on chromosome 8. Black arrows indicate peak tip SNPs and dashed arrows indicate SNPs included in haplotype analysis (listed in Fig 3). Numbers in boxes indicate R^2^ values between each two markers. Dark black boxes without numbers have R^2^ = 1.
(TIF)

S8 Fig Linkage disequilibrium (LD) analysis for loci associated with grain P concentration at 2.57–2.73 Mb on chromosome 11. Black arrows indicate peak tip SNPs and dashed arrows indicate SNPs included in haplotype analysis (listed in Fig 3). Numbers in boxes indicate R^2^ values between each two markers. Dark black boxes without numbers have R^2^ = 1.
(TIF)

S9 Fig Linkage disequilibrium (LD) analysis for loci associated with grain P concentration at 3.38–3.49 Mb on chromosome 12. Black arrows indicate peak tip SNPs and dashed arrows indicate SNPs included in haplotype analysis (listed in Fig 3). Numbers in boxes indicate R^2^ values between each two markers. Dark black boxes without numbers have R^2^ = 1.
(TIF)

S10 Fig Linkage disequilibrium (LD) analysis for loci associated with grain P concentration at 8.37–8.92 Mb on chromosome 12. Black arrows indicate peak tip SNPs and dashed arrows indicate SNPs included in haplotype analysis (listed in Fig 3). Numbers in boxes indicate R^2^ values between each two markers. Dark black boxes without numbers have R^2^ = 1.
(TIF)

S1 Table Partial correlations with grain P concentrations when controlled for variation in grain yield (GY) or total aboveground plant P (TP). (TIF)
