# Supplementary figures and images for: Can natural variation in grain P concentrations be exploited in rice breeding to lower fertilizer requirements?
Source: PLoS One. 2017 Jun 26;12(6):e0179484. doi: 10.1371/journal.pone.0179484 (PMC5484489; doi:10.1371/journal.pone.0179484)

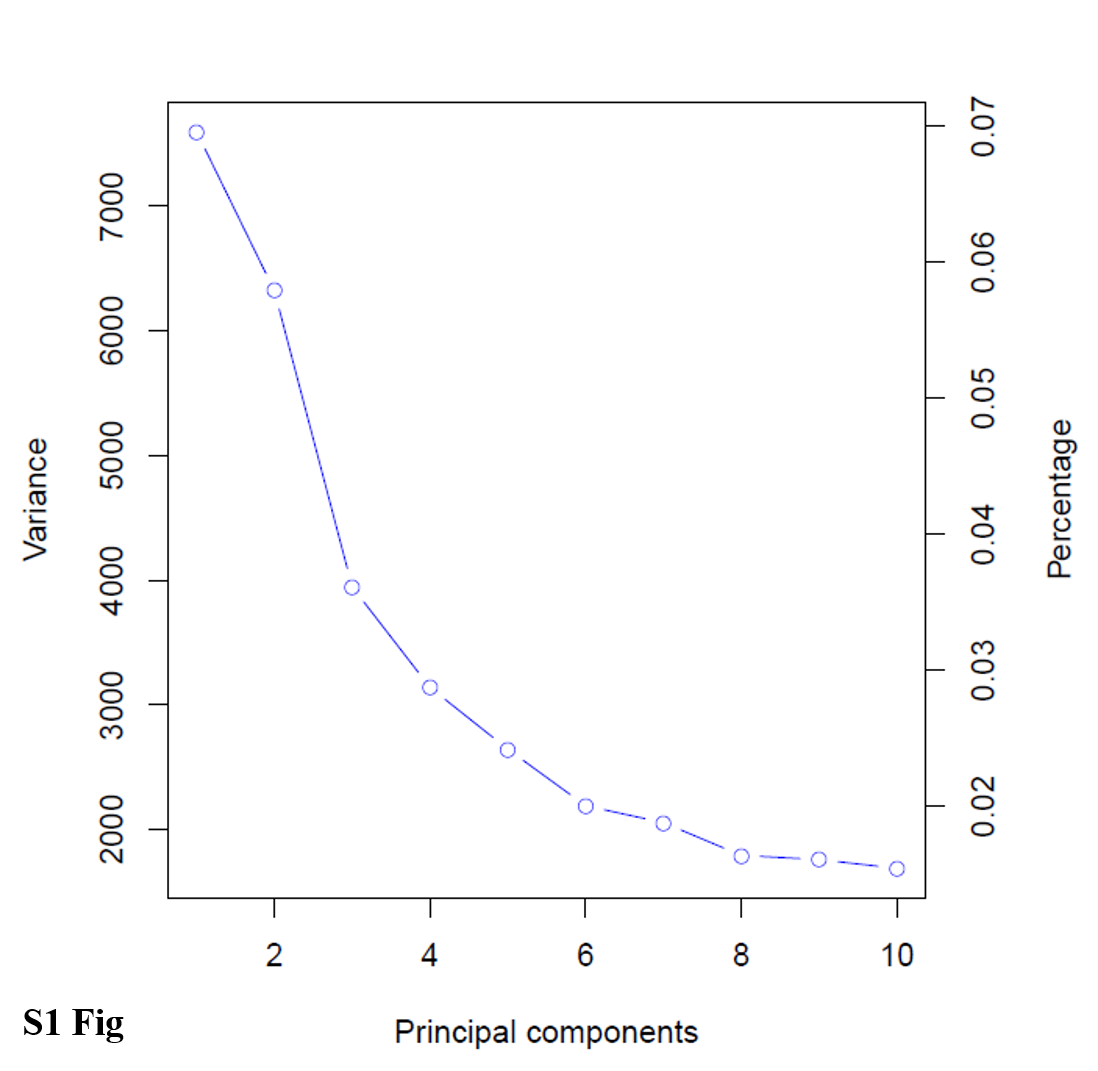

Supplement: S1 Fig — (TIF) [file pone.0179484.s001.tif]

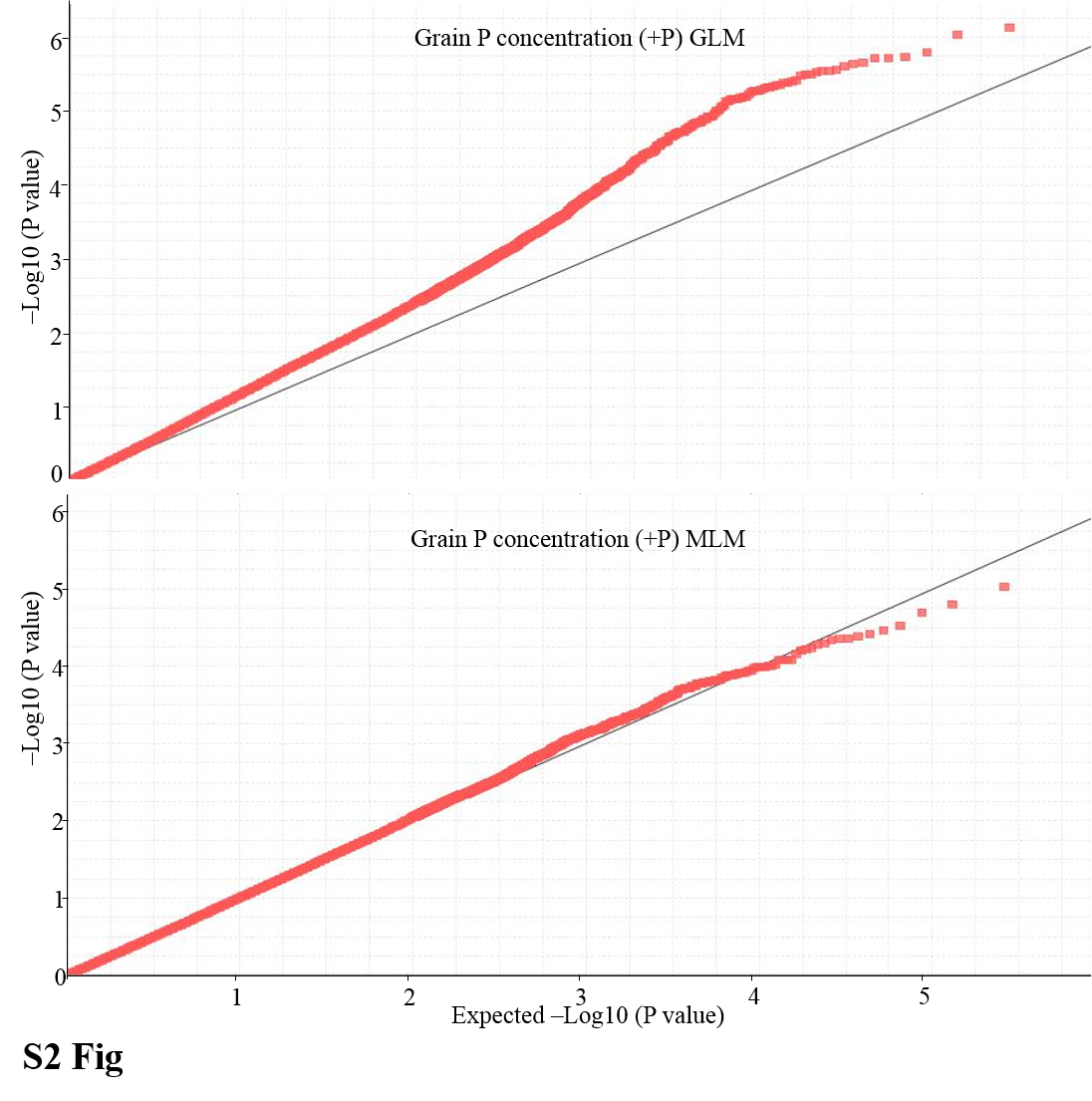

Supplement: S2 Fig — (TIF) [file pone.0179484.s002.tif]

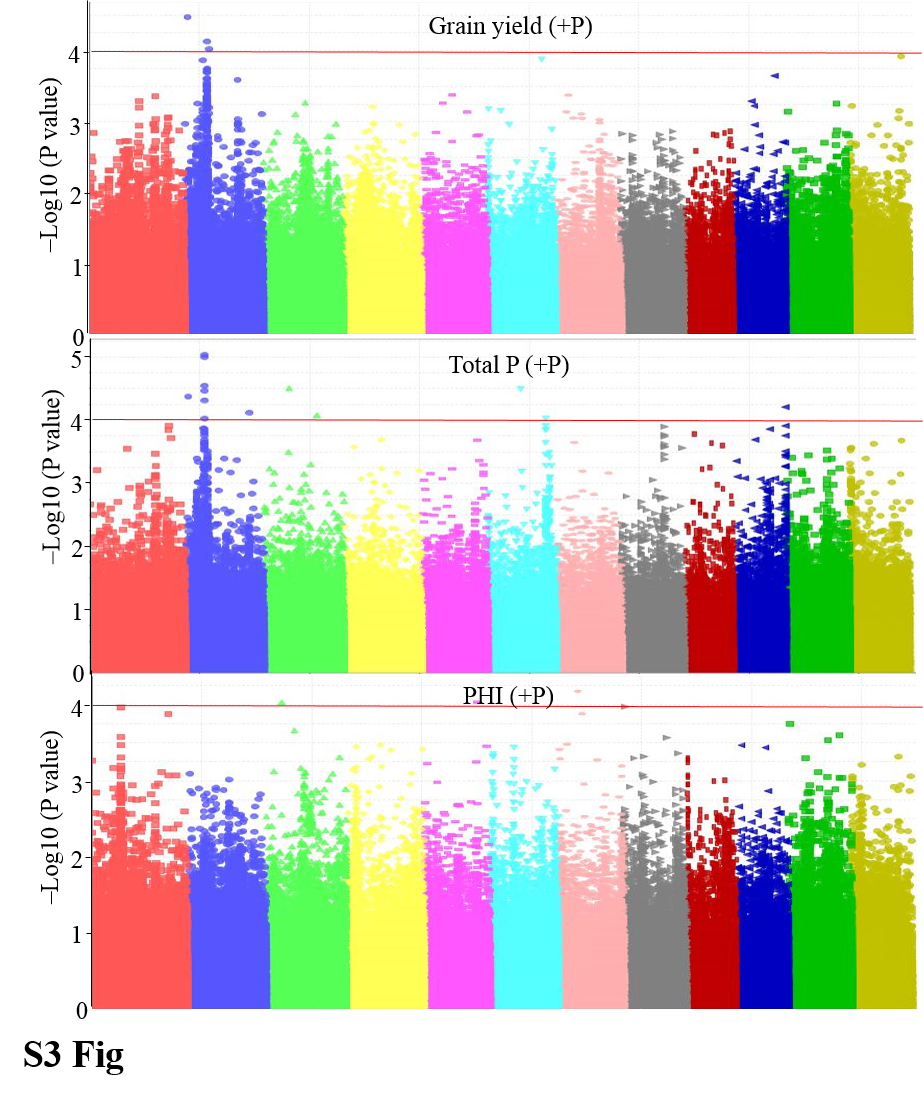

Supplement: S3 Fig — (TIF) [file pone.0179484.s003.tif]

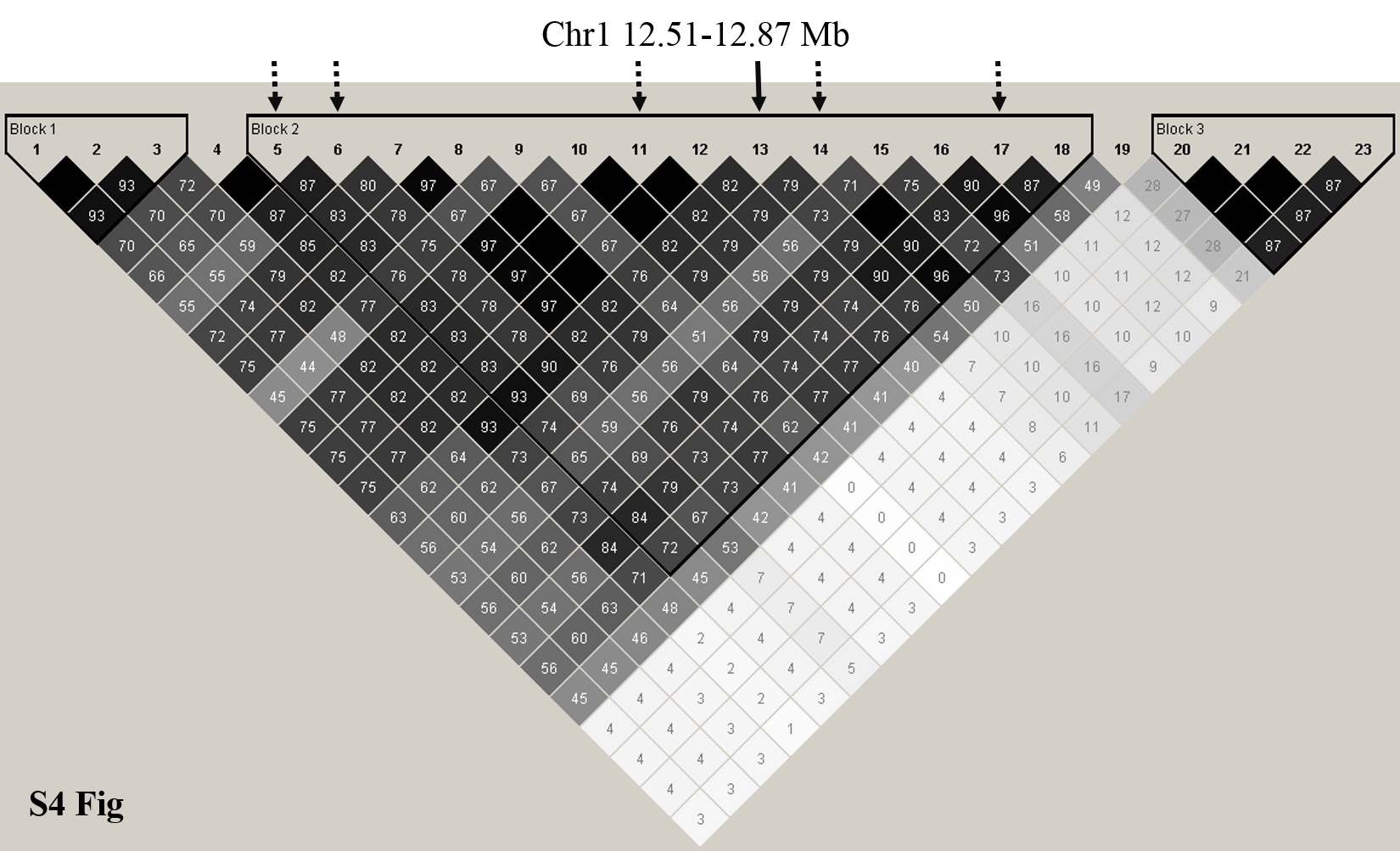

Supplement: S4 Fig — Black arrows indicate peak tip SNPs and dashed arrows indicate SNPs included in haplotype analysis (listed in Fig 3). Numbers in boxes indicate R2 values between each two markers. Dark black boxes without numbers have R2 = 1. (TIF) [file pone.0179484.s004.tif]

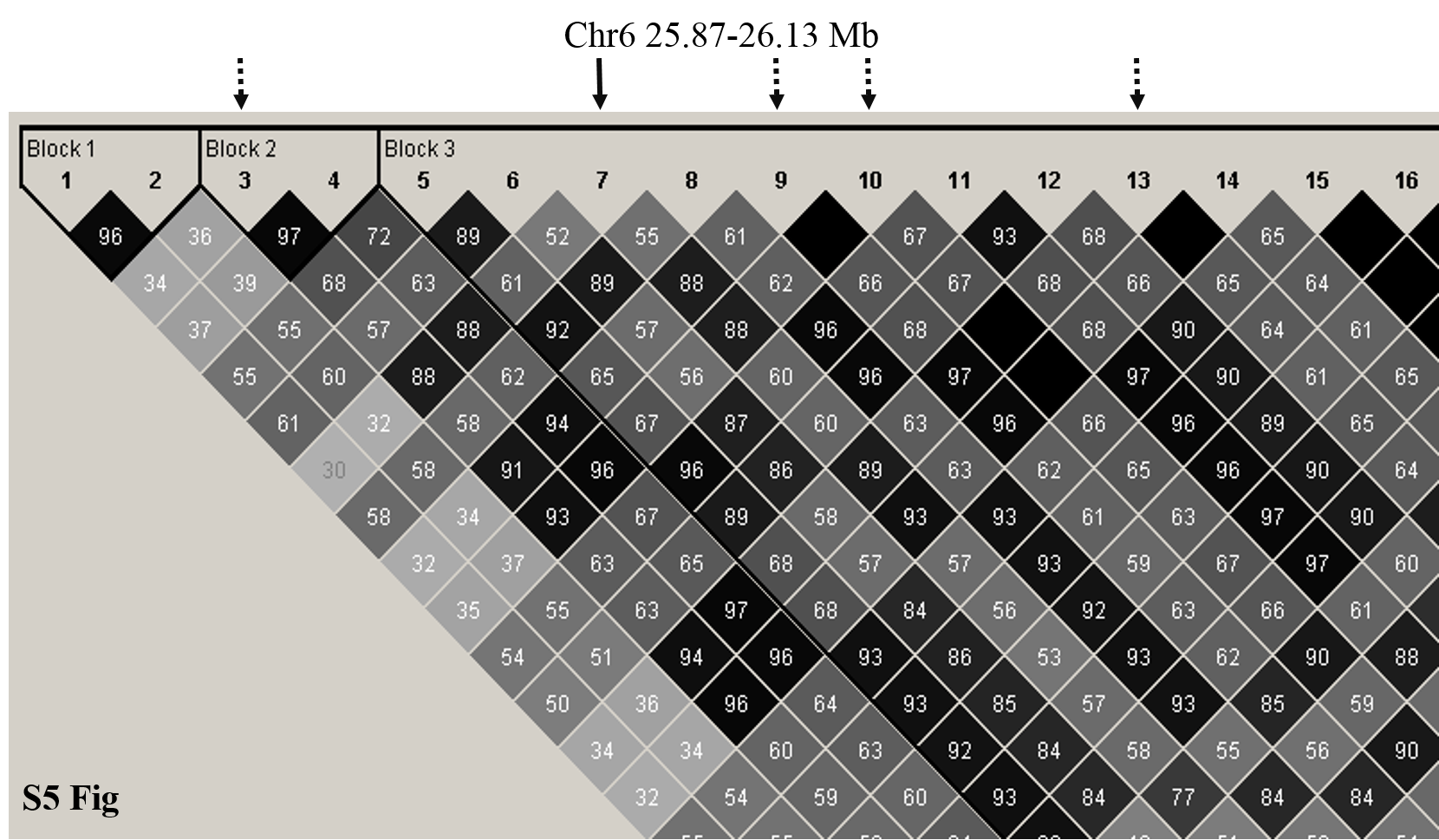

Supplement: S5 Fig — Black arrows indicate peak tip SNPs and dashed arrows indicate SNPs included in haplotype analysis (listed in Fig 3). Numbers in boxes indicate R2 values between each two markers. Dark black boxes without numbers have R2 = 1. (TIF) [file pone.0179484.s005.tif]

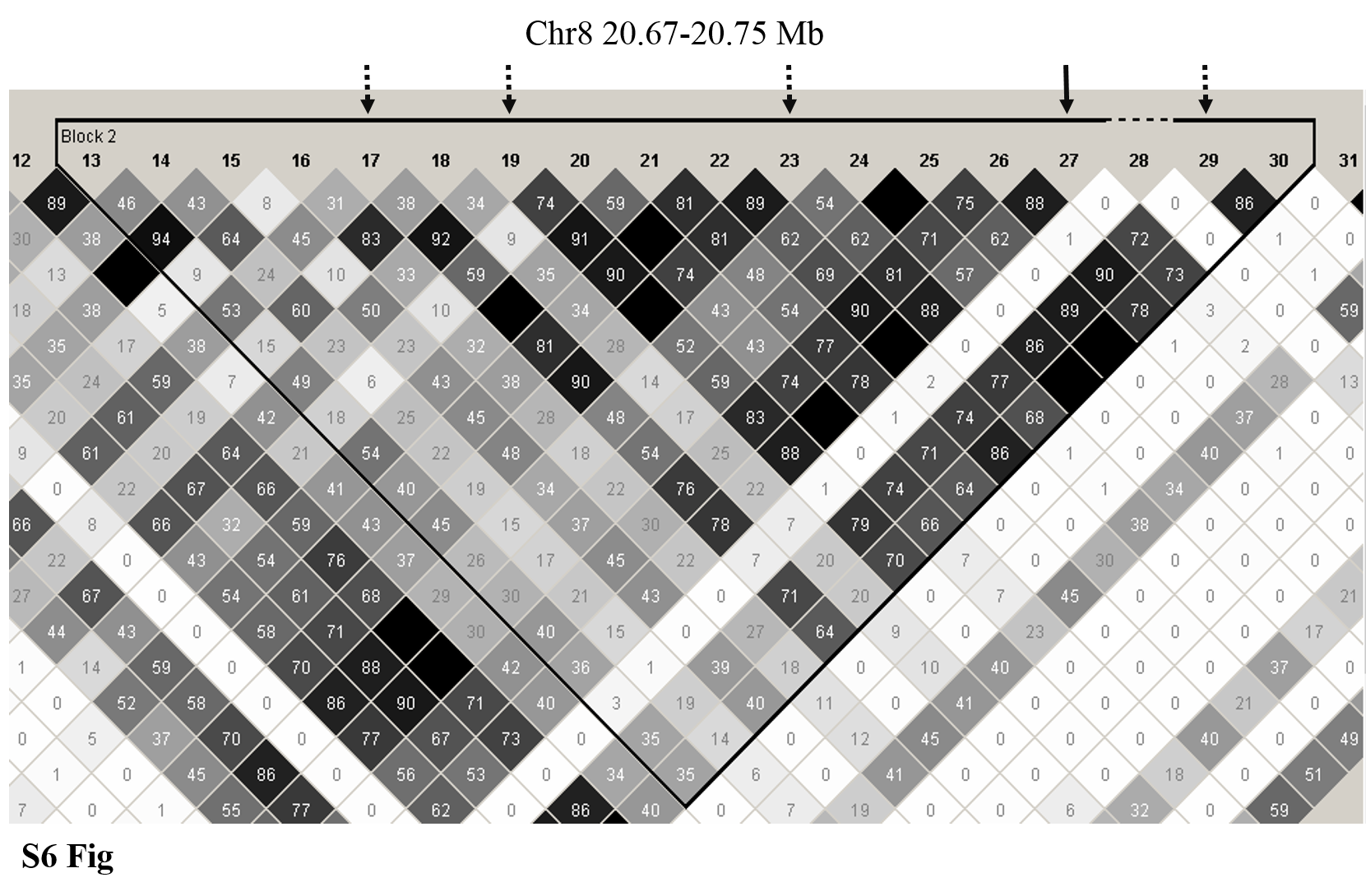

Supplement: S6 Fig — Black arrows indicate peak tip SNPs and dashed arrows indicate SNPs included in haplotype analysis (listed in Fig 3). Numbers in boxes indicate R2 values between each two markers. Dark black boxes without numbers have R2 = 1. (TIF) [file pone.0179484.s006.tif]

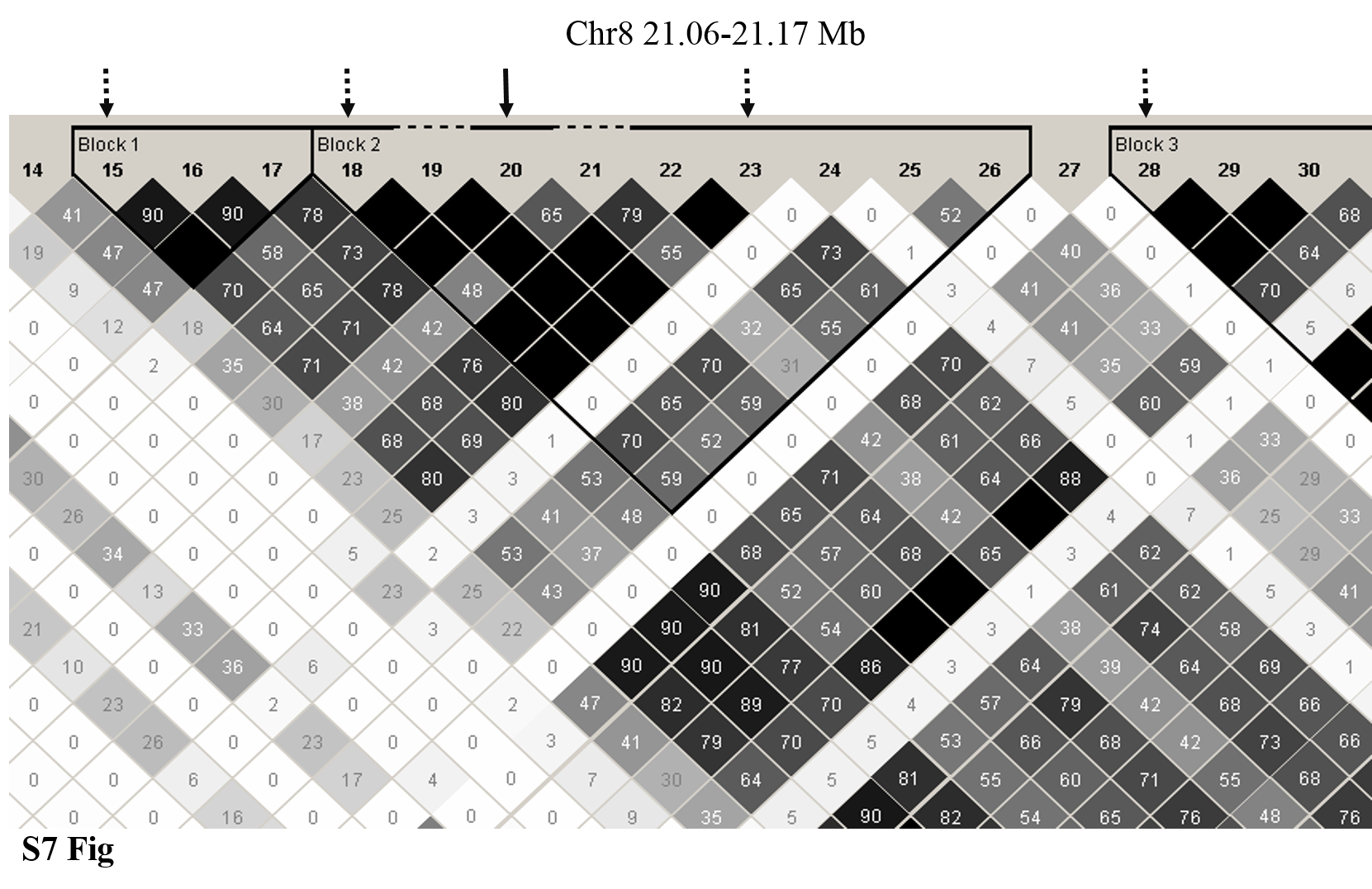

Supplement: S7 Fig — Black arrows indicate peak tip SNPs and dashed arrows indicate SNPs included in haplotype analysis (listed in Fig 3). Numbers in boxes indicate R2 values between each two markers. Dark black boxes without numbers have R2 = 1. (TIF) [file pone.0179484.s007.tif]

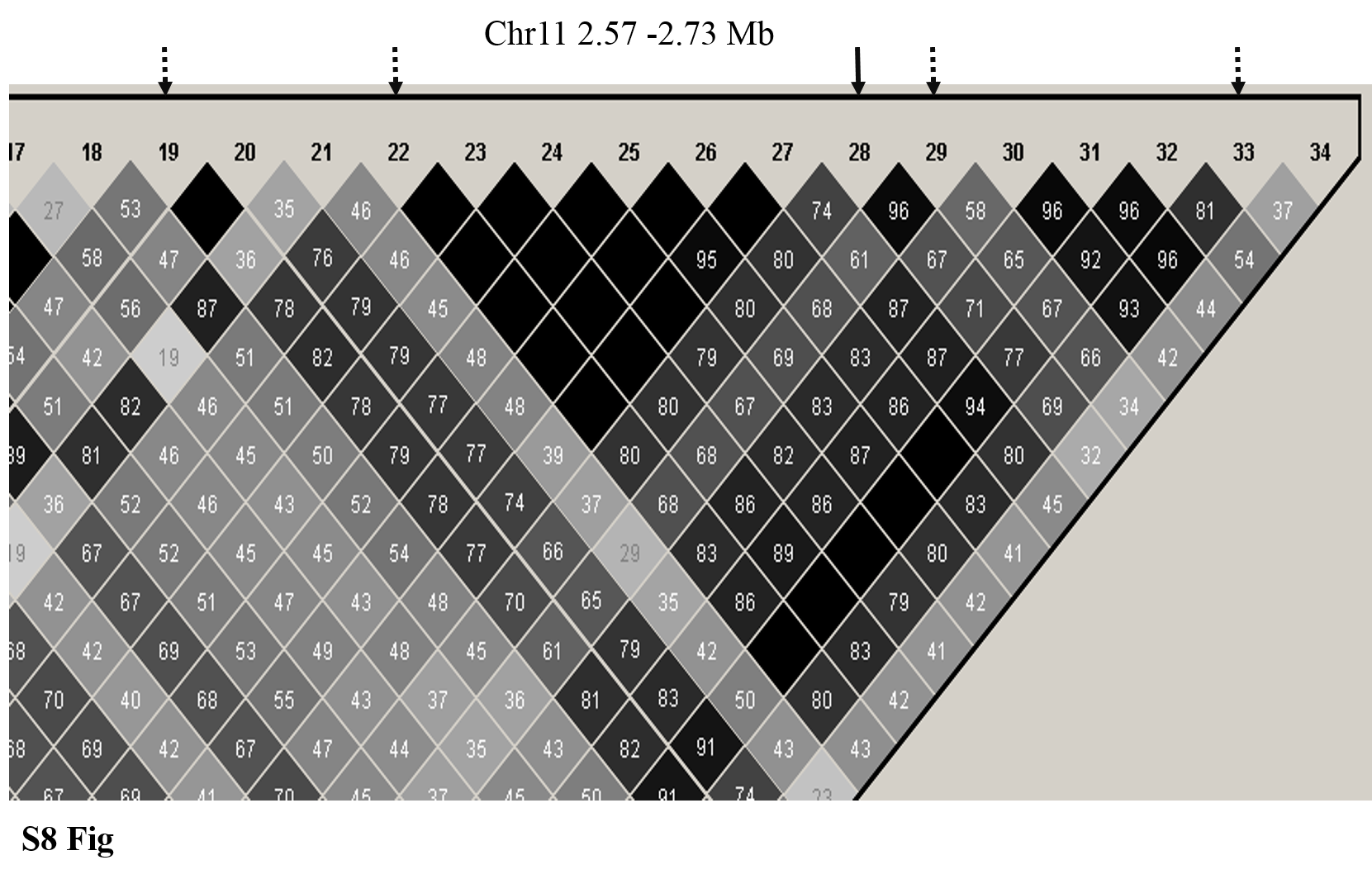

Supplement: S8 Fig — Black arrows indicate peak tip SNPs and dashed arrows indicate SNPs included in haplotype analysis (listed in Fig 3). Numbers in boxes indicate R2 values between each two markers. Dark black boxes without numbers have R2 = 1. (TIF) [file pone.0179484.s008.tif]

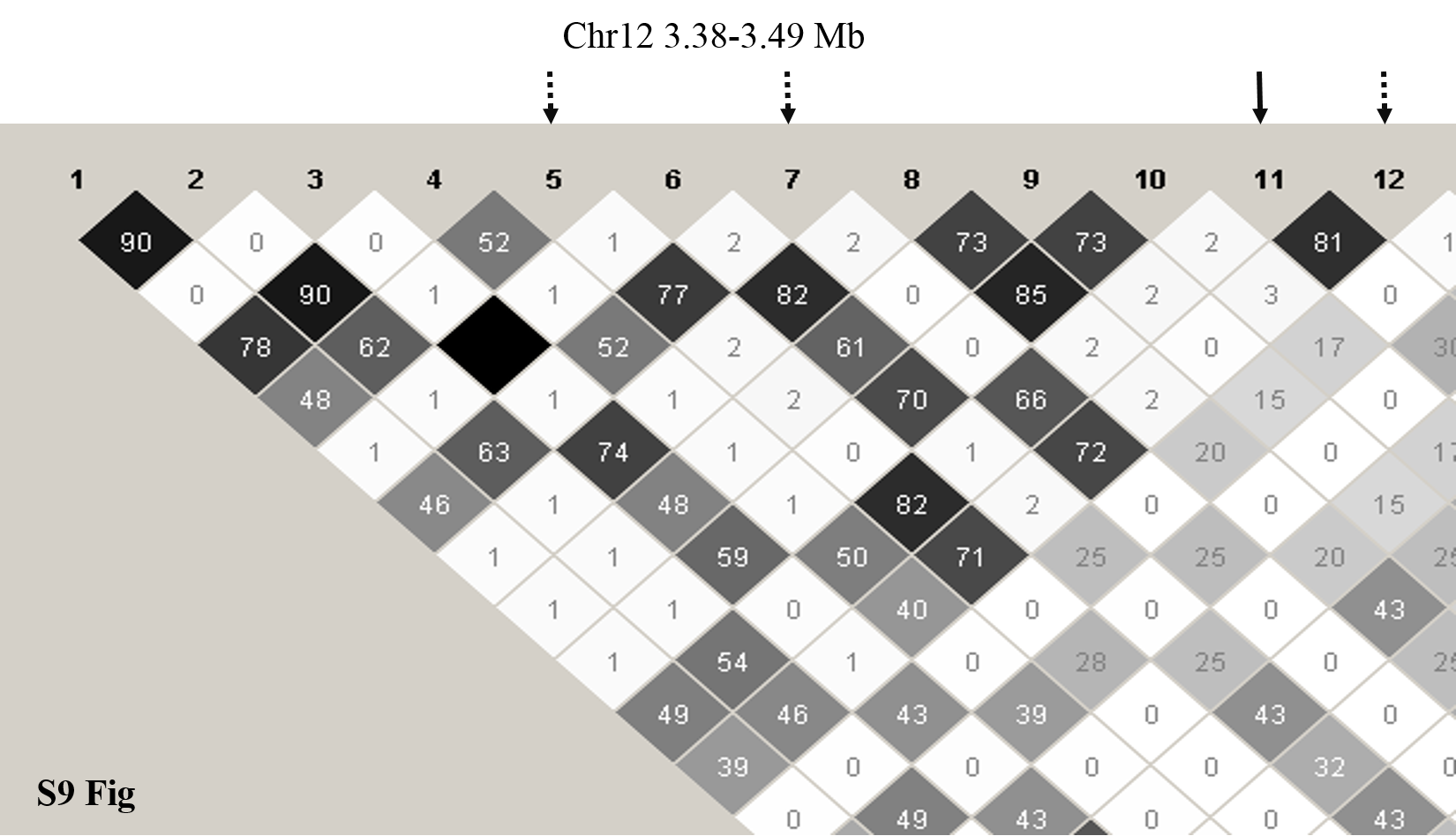

Supplement: S9 Fig — Black arrows indicate peak tip SNPs and dashed arrows indicate SNPs included in haplotype analysis (listed in Fig 3). Numbers in boxes indicate R2 values between each two markers. Dark black boxes without numbers have R2 = 1. (TIF) [file pone.0179484.s009.tif]

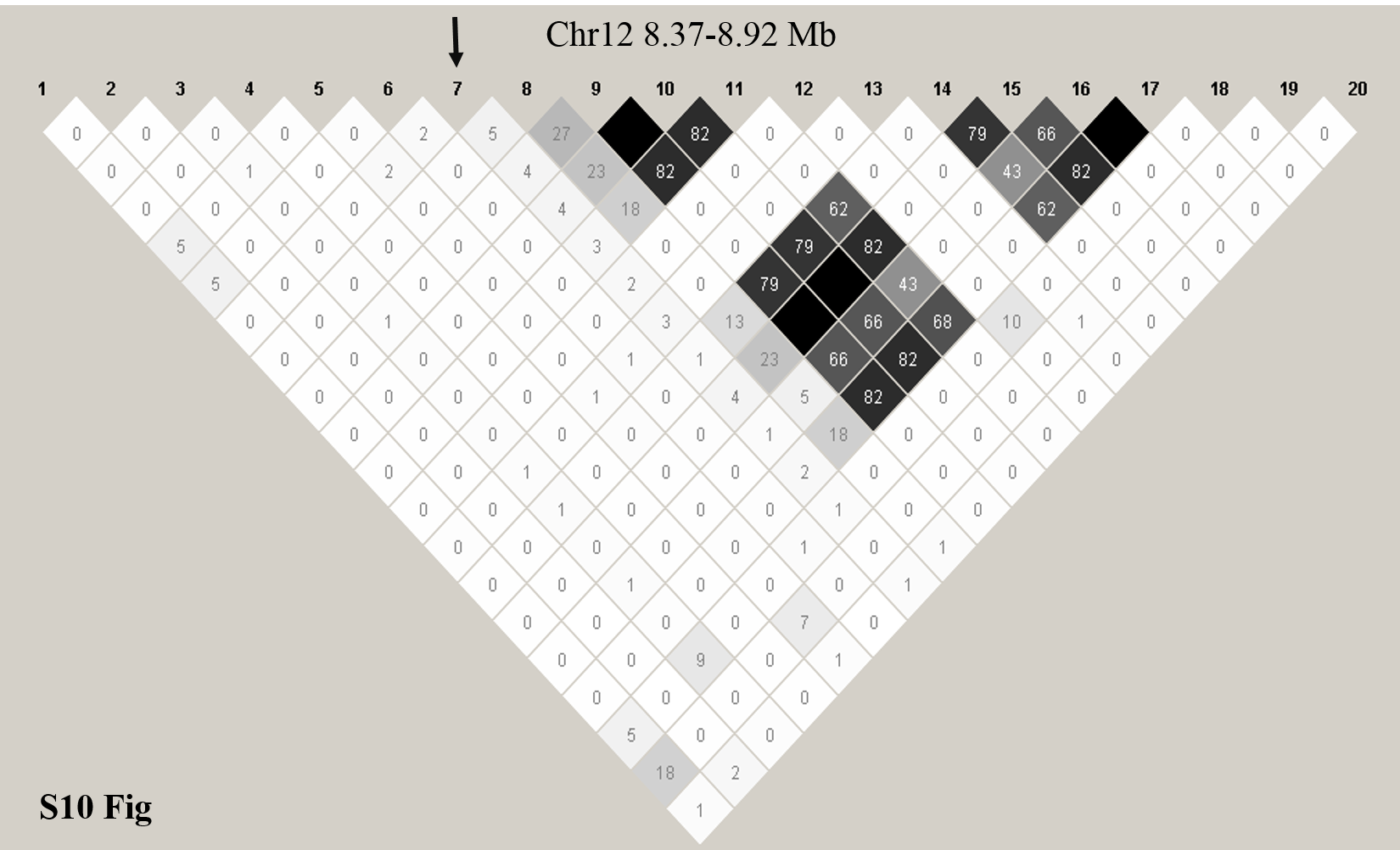

Supplement: S10 Fig — Black arrows indicate peak tip SNPs and dashed arrows indicate SNPs included in haplotype analysis (listed in Fig 3). Numbers in boxes indicate R2 values between each two markers. Dark black boxes without numbers have R2 = 1. (TIF) [file pone.0179484.s010.tif]

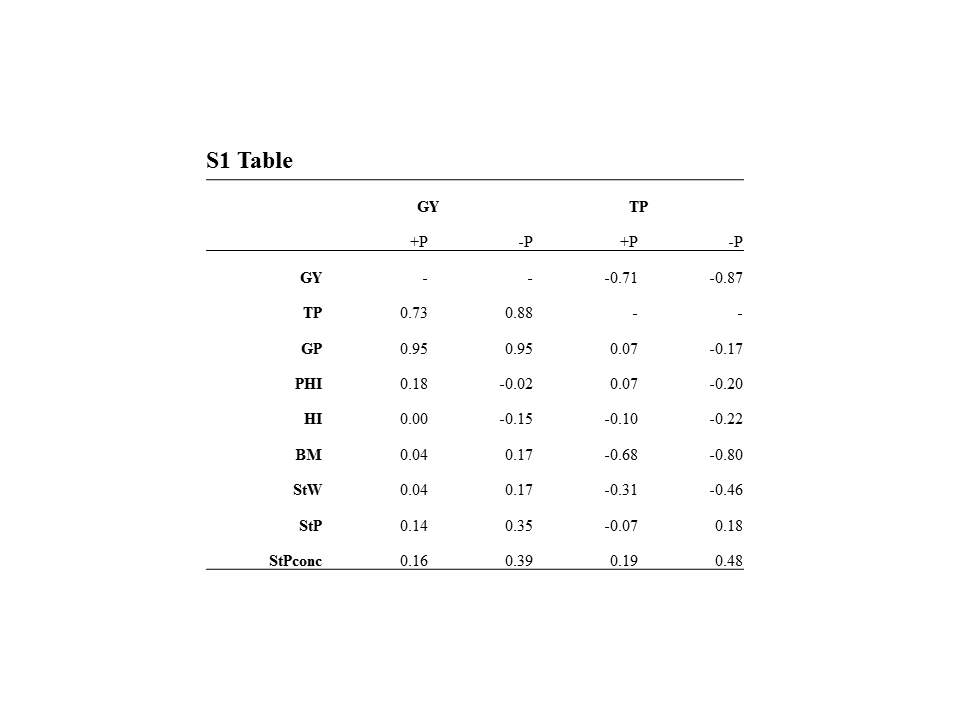

Supplement: S1 Table — (TIF) [file pone.0179484.s011.tif]
